# The incidence of avid lesions in head and neck cancer patients undergoing positron emission tomography-computed tomography scanning

**DOI:** 10.1017/S0022215125103976

**Published:** 2026-02

**Authors:** Abbie Carter, Huw Rhys Davies, Ali A. Salamat, Neela Mouli Doddi

**Affiliations:** 1 Cardiff University School of Medicine, Wales, UK; 2 ENT Department, Royal Glamorgan Hospital, Llantrisant, Wales, UK

**Keywords:** carcinoma, squamous cell, head and neck neoplasms, neoplasms, unknown primary, positron emission tomography-computed tomography, retrospective studies

## Abstract

**Objectives:**

This study aimed to determine the incidence, location and outcome of incidental avid lesions on positron emission tomography-computed tomography scans for head and neck cancer.

**Methods:**

A retrospective study reviewing digital case notes, performed from a single centre. Clinicopathological information was collected and incidental avid lesions on positron emission tomography-computed tomography reports were recorded. Further investigations were followed up to determine the outcome of the lesions.

**Results:**

A total of 281 patients undergoing staging positron emission tomography-computed tomography (stages T4, N3 or unknown primary) and/or treatment response positron emission tomography-computed tomography scans for head and neck cancer were identified, with 363 incidental avid lesions reported in 369 scans. The most common location was the abdomen (30.0 per cent), followed by thorax (28.9 per cent). A total of 33.1 per cent of lesions had further investigation. The rate of incidental synchronous primary was 3.6 per cent.

**Conclusion:**

The benefit of investigating carefully selected incidental avid lesions outweighs the harm of investigation, as it may alter management. There is a need for a standardised pathway for investigating these lesions in head and neck cancer services.

## Introduction

Head and neck cancer (HNC) is the seventh most common cancer worldwide and incidence continues to increase, with a predicted 30 per cent rise (1.08 million new cases per year) by 2030.^1^ Naturally, the use of positron emission tomography-computed tomography (PET-CT) for assessment of HNC is increasing with this rising incidence. PET-CT has a valuable role in diagnosis, staging and assessing treatment response, with a high sensitivity for detecting HNC.^2^ It is a whole-body, combined imaging technique, providing anatomical (CT) and metabolic (PET) information. Ultimately, full body scanning introduces the chance of avidity elsewhere, which can be false positive (glucose uptake from inflammation, infection and scarring), but occasionally, represents a synchronous malignancy. This raises a question of whether to investigate these incidental avid lesions. There is a balance between the potential harm of over-investigating benign false-positive lesions and the need to identify true-positive synchronous primaries, which may alter management. This study aims to determine the incidence, location, investigation and clinical outcome of avid lesions on PET-CT scans for HNC patients.

## Materials and methods

This was a retrospective study of patients presenting to the HNC service over an eight-year period (2013–2021). Our service is situated in a UK district general hospital, covering a large population across South Wales.

Inclusion criteria: Patients who were investigated with PET-CT either as part of initial staging work-up or for monitoring of treatment disease response were included. In line with NHS Wales Joint Commissioning Committee (NWJCC) guidelines, in Wales, staging (pre-treatment) PET-CT is only offered to T4 oropharyngeal, nasopharyngeal and hypopharyngeal squamous cell carcinoma (SCC); N3 disease of the upper aerodigestive tract; and patients with clinical suspicion of head and neck SCC of unknown primary.^3^ Treatment response (post-treatment) PET-CT is offered to patients with locally advanced, node positive HNC to assess response three to six months after completion of primary non-surgical treatment (radical radiotherapy +/- chemotherapy). Therefore, it should be noted in our population, there are a high number of treatment response PET-CT scans, and only T4 staged lesions would have had pre- and post-treatment PET-CT.^3^ Post-treatment PET-CT is not indicated after surgical treatment, as per NWJCC guidelines.

Exclusion criteria: Patients were excluded if they had a primary thyroid malignancy or if the PET-CT scan (staging or treatment response) was unavailable.

Digital case notes were reviewed to extract clinicopathological characteristics, including age at diagnosis, sex, primary site, histological diagnosis and ultimately the tumour, node, metastasis (TNM) staging of their malignancy and p16 status if available. PET-CT scans were reported by a specialist head and neck radiologist and the location of each avid lesion was recorded. Incidental avid lesions were defined as any increased avidity deemed unrelated, or indeterminate, to the confirmed primary site or regional neck lymph node spread.

The PET-CT report and case notes were interrogated to determine whether these lesions were investigated or not, and the clinical decision-making process. If investigated, the specific test(s) done and whether they were invasive diagnostic (e.g., biopsy, endoscopy, blood tests) or non-invasive diagnostic (e.g., imaging, clinical review) were recorded. The clinicopathological outcome of each lesion was documented and assigned a category: benign, malignant (synchronous primary or site of previously unknown primary) or metastatic. Ethical approval for this project was not required.

## Results and analysis

### Population and demographics

327 HNC patients’ case notes were reviewed; 46 patients were deemed ineligible based on the exclusion criteria. Therefore, the final study included 281 patients with at least one PET-CT with a full report accessible; 88 of these patients (31.3 per cent) had staging and treatment response PET-CT scans, with the remaining 193 only having a treatment response PET-CT. A total of 369 PET scans were analysed, with a mean of 1.31 scans per patient.

The mean age of those undergoing PET-CT was 61 years (range 36–84), and 220 (78.3 per cent) of these patients were male. A total of 287 (100 per cent) of tumours were histologically reported as squamous cell carcinoma (SCC), the most common type of HNC, including cancer of the unknown primary (CUP), where the neck node(s) were histologically SCC.^4^ The predominant tumour location was oropharynx (82.2 per cent), with all locations shown in Table 1A and the subsites of patients with two primary head and neck tumours shown in Table 1B.

**Table 1A.** Location of primary head and neck tumours amongst the study population

Tumour location	Frequency	Percentage
Oropharynx	236	82.2%
Hypopharynx	20	7.0%
Larynx	17	5.9%
Cancer of unknown primary	7	2.4%
Nasopharynx	4	1.4%
Sinonasal	3	1.0%
Total	287	

*Note:* Six patients had two primary tumour sub-sites; of these, all had at least one oropharyngeal primary site, the second primary sub-sites were oropharynx (two), larynx (two) and hypopharynx (two).

**Table 1B.** Location of subsites in patients with two primary head and neck tumours

Patient	Primary number 1	Primary number 2
1	Oropharynx (R tonsil)	Oropharynx (L tonsil)
2	Oropharynx (R tonsil)	Oropharynx (tongue base)
3	Oropharynx (tonsil)	Larynx (supraglottis)
4	Oropharynx (R tonsil)	Larynx (aryepiglottic fold)
5	Oropharynx (tongue base)	Hypopharynx
6	Oropharynx	Hypopharynx

Amongst 236 SCCs of the oropharynx; 184 (78.0 per cent) were p16 positive, 42 were negative and 10 were not documented. Seven (2.4 per cent) patients were concluded to have cancer of the unknown primary (CUP) after PET-CT imaging and/or surgical biopsy, and five of these were p16 positive.

### Incidence of lesions

**Figure 1.**
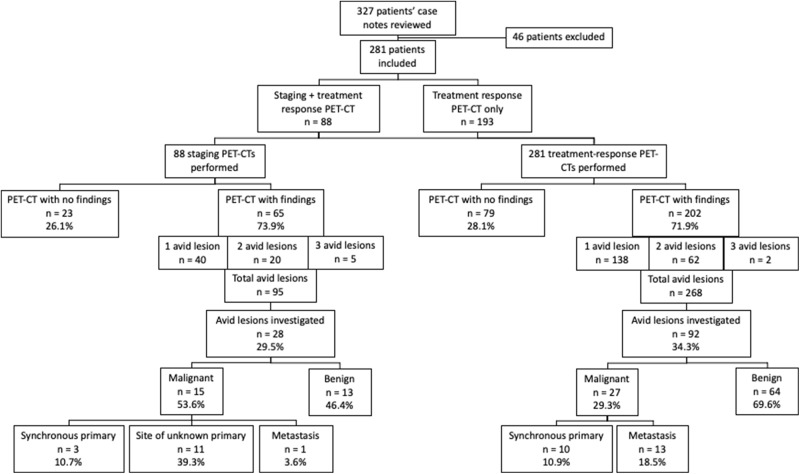
Flow chart of incidental avid lesions by investigation pathway.

A total of 363 incidental avid lesions were identified across 369 scans. Figure 1 summarises the number of incidental avid lesions found, further investigation and final pathological classification. The rate of incidental avid lesions reported in the staging PET-CT was 1.1 per scan, versus 0.95 per scan for response PET-CT. The number of lesions in each location, across staging and response PET-CT scans, are summarised in Table 2.

**Table 2.** Location of incidental avid lesions on PET-CT scans

Location	Avid lesions - staging PET-CT, n (%)	Avid lesions - response PET-CT, n (%)	Total avid lesions	Overall percentage
Abdomen and GI tract	23 (24.2%)	86 (32.1%)	109	30.0%
Thorax	17 (17.9%)	88 (32.8%)	105	28.9%
Head and neck	37 (38.9%)	29 (10.8%)	66	18.2%
Musculoskeletal	5 (5.3%)	31 (11.6%)	36	9.9%
Thyroid	6 (6.3%)	19 (7.1%)	25	6.9%
Genitourinary	5 (5.3%)	8 (3.0%)	13	3.6%
Other	2 (2.1%)	7 (2.6%)	9	2.5%
Total	95	268	363	

GI = gastrointestinal; PET-CT = positron emission tomography-computed tomography

### Anatomical sites of avid lesions

The most common location for incidental avid lesions overall was the abdomen and gastrointestinal (GI) tract (*n* = 109; 30.0 per cent), followed by thorax (*n* = 105; 28.9 magnetic resonance imaging [MRI]). Sixty-six lesions were related to the head and neck (18.2 per cent), 36 to the musculoskeletal system (9.9 per cent), 25 to the thyroid (6.9 per cent), 13 to the genitourinary system (3.6 per cent) and 9 in other locations (2.5 per cent). In staging PET, the commonest location for incidental avid lesions was head and neck (*n* = 37; 38.9 per cent). In response PET, the commonest location was thorax (*n* = 88; 32.8 per cent), closely followed by abdomen and GI tract (*n* = 86; 32.1 per cent). Whilst confident in the robust data set of our population studied, the retrospective nature of this study precludes accurately identifying whether lesions were pre-treatment or post-treatment, but these lesions weren’t demonstrated on previous or complimentary imaging modalities (ultrasound, CT and MRI).

**Figure 2.**
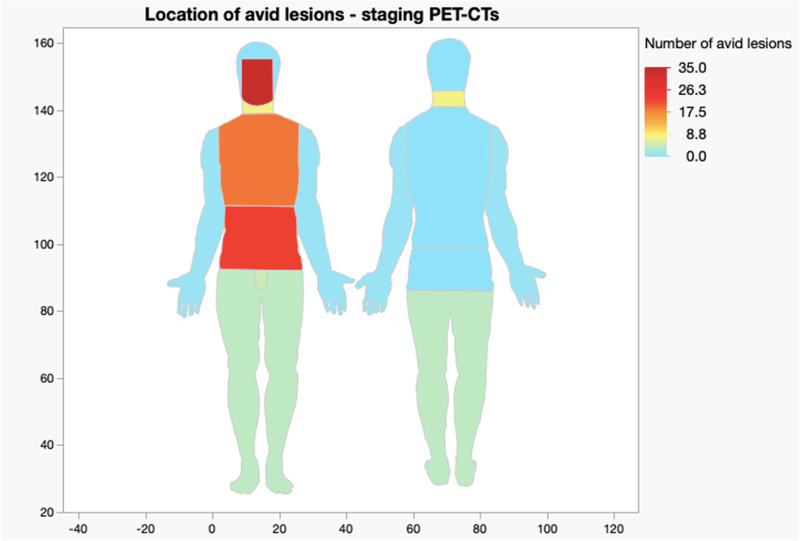
Heat map showing location and frequency of avid lesions in staging PET-CT scans.^5^^,^^6^

**Figure 3.**
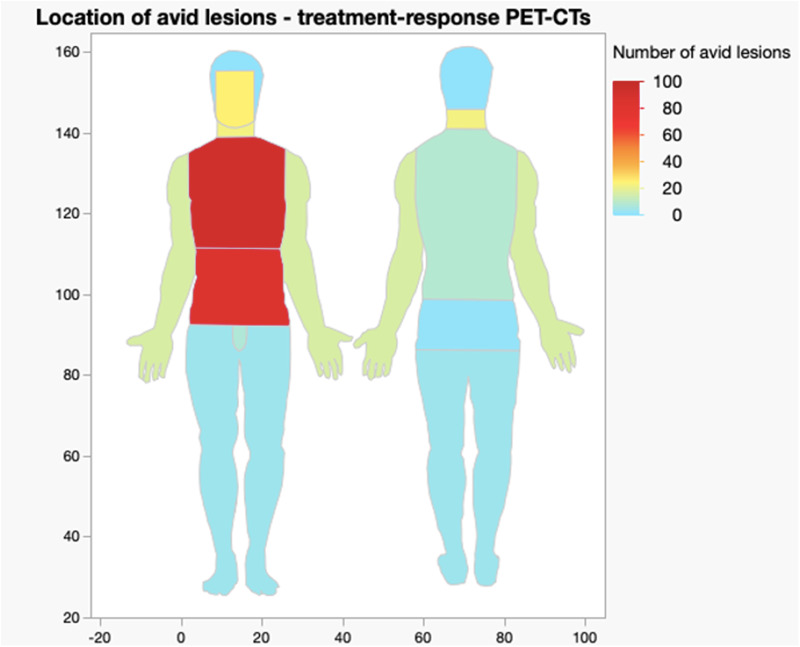
Heat map showing location and frequency of avid lesions in treatment-response PET-CT scans.^5^^,^^6^

A visual breakdown of location and frequency is demonstrated in the heat maps in Figures 2 and 3.^5^^,^^6^

### Investigations undertaken

A total of 120 (33.1 per cent) incidental avid lesions were investigated further, 28 (29.5 per cent) in the staging PET and 92 (34.3 per cent) in the response PET, with a total of 142 tests (some lesions had multiple separate investigations). Most common radiologist comments dismissing the need for further investigation were ‘physiological’, ‘inflammatory’ and ‘doubtful significance’. Seven patients who underwent treatment response PET-CT had incidental lesions on their PET-CT which were not investigated further due to the patients being made palliative and deemed unfit for further investigation following PET-CT. This cohort of patients died between 14 days and 6 months of the treatment-response PET-CT which found these lesions. Common reasons for recommending investigations were ‘potential incidental primary’, ‘uncertain significance’ and ‘potential metastasi’. Tables 3 and 4 elaborate on the type of tests carried out for lesions in staging and response PET-CT scans, respectively. Out of 34 investigations done for 28 incidental avid lesions on staging PET-CT, 26 (76.5 per cent) were invasive diagnostic tests versus 8 (23.5 per cent) ‘non-invasive’ tests. Out of 108 investigations done for 92 incidental avid lesions on treatment response PET-CT, 55 (57.0 per cent) were invasive and 53 (43.0 per cent) were non-invasive.

**Table 3.** Further investigations done for incidental avid lesions found on staging PET-CT

Location	No. of lesions investigated	No. of tests	Invasive tests*	Non-invasive tests*
Head and neck	14	15	Biopsy (13)	US, MRI
Thyroid	4	5	US FNA (3), biopsy, TFT	
Abdomen and GI tract	4	4	Colonoscopy + biopsy (2), biopsy	Clinical review
Genitourinary	2	5	PSA (2), MRI-guided biopsy	MRI (2)
Other	2	3	US-guided biopsy	Mammogram, US
Thorax	2	2	EBUS-TBNA	CT
Musculoskeletal	0	0		
Total	28	34	26	8

Abbreviations: CT = computed tomography; EBUS-TBNA = endobronchial ultrasound-guided transbronchial needle aspiration; MRI = magnetic resonance imaging; PSA = prostate-specific antigen; TFT = thyroid Function fest; US FNA = ultrasound fine needle aspiration.

* Number in parentheses denotes frequency of an investigation, otherwise *n* = 1.

**Table 4.** Further investigations done for incidental avid lesions found on response PET-CT

Location	No. of lesions investigated	Total no. of tests	Invasive tests*	Non-invasive tests*
Thorax	34	36	EBUS-TBNA (3), biopsy, bronchoscopy, surgical resection	CT (29), chest X-ray
Abdomen and GI tract	26	28	Colonoscopy + biopsy (16), biopsy, endoscopy + biopsy, dexamethasone suppression test	US (3), MRI (2), CT (2), urinary free cortisol, clinical review
Thyroid	12	14	US FNA (5), TFT (7)	US (2)
Musculoskeletal	7	10	Biopsy (4)	MRI (5), CT
Head and neck	6	7	Biopsy (3), US FNA (2)	CT, clinical review
Genitourinary	5	11	PSA (4), MRI-guided biopsy (2), Ca−125	MRI (4)
Other	2	2	Surgical excision, US FNA	
Total	92	108	55	53

Abbreviations: CT = computed tomography; EBUS-TBNA = endobronchial ultrasound-guided transbronchial needle aspiration; MRI = magnetic resonance imaging; PSA = prostate-specific antigen; TFT = thyroid Function fest; US FNA = ultrasound fine needle aspiration.

* Number in parentheses denotes frequency of an investigation, otherwise *n* = 1.

### Clinicopathological outcomes

Of 28 staging PET avid lesions investigated, 13 (46.4 per cent) were benign, 3 (10.7 per cent) identified a previously undiagnosed synchronous primary, 11 (39.3 per cent) identified a previously unknown location of HNC primary and 1 (3.6 per cent) was metastasis of the primary tumour (SCC to the thyroid). Of 92 response PET avid lesions investigated, 65 (70.7 per cent) were benign, 10 (10.9 per cent) identified a previously undiagnosed synchronous primary and 17 (18.5 per cent) showed metastasis of the primary tumour.

Benign incidental lesions were found in the abdomen (26, including polyps, an abdominal wall neuroma, adrenal phaeochromocytoma, liver haemangioma and inflammatory changes including panniculitis and chronic oesophagitis); thorax (11, including resolved inflammatory changes and fibrosis); thyroid (12, including thyroiditis and a Thy1 lesion not followed up); head and neck (8, including a Warthin’s tumour, Tornwaldt cyst, sialadenitis and radiotherapy changes); genitourinary system (4, including prostatic hyperplasia); musculoskeletal system (3, including a benign enchondroma of the humerus); breast (1); mammary nodes (1); reactive axillary nodes (1) and skin (1, a dermatofibroma).

Notably, the patient with previously undiagnosed pheochromocytoma had resection of the tumour for symptomatic relief, despite its benign nature. The dermatofibroma was excised with diagnostic and therapeutic intent. Patients with newly diagnosed primary hypothyroidism were commenced on thyroxine.

The rate of synchronous primaries identified from incidental avid lesions on all PET-CT scans was 3.6 per cent (13 lesions out of 363). The rate of synchronous primaries on staging PET-CT was 3.2 per cent (3 out of 95) and from response PET-CT was 3.7 per cent (10 out of 268). Previously undiagnosed synchronous primaries were found in the lung (four, adenocarcinoma and SCC), large bowel (three, adenocarcinoma), thyroid (three, papillary carcinoma) and prostate (three, adenocarcinoma). One patient had two synchronous primaries diagnosed from PET-CT and subsequent investigations: papillary thyroid carcinoma and lung adenocarcinoma.

## Discussion

### Key findings

Almost three-quarters (72.4 per cent) of patients undergoing PET-CT for staging or treatment response had at least one incidental finding on their scan(s). Around a third (33.1 per cent) of incidental avid lesions found had further investigation, introducing implications for the patient and health service beyond the initial PET-CT. First, there is additional financial cost, plus resource and time taken for referrals to specialties for invasive tests (such as colonoscopy or endobronchial ultrasound-guided transbronchial needle aspiration [EBUS-TBNA]), often compounded by considerable waiting lists. Second, the psychological burden of further investigations for patients already undergoing cancer diagnostics and treatment cannot be underestimated. The economic and psychological impacts of investigation in this specific HNC population require further qualification in future studies.

In our study, 65.0 per cent of all incidental avid lesions investigated turned out to be benign (false positives), towards the higher end of a range of false-positive rates seen in other studies, including Beatty *et al.* (43 per cent for all investigated primaries) and Casselden *et al*. (53.1 per cent).^7^^,^^8^ If there are too many false positives, the potential ‘harm’ of investigating incidental lesions may surpass the benefit.^9^ However, our study shows that PET-CT scanning also identified 13 true positives (previously undiagnosed synchronous primaries). These were often early stage (T1/2N0) and highly treatable, if identified promptly whilst the patient is undergoing close clinical work-up or follow-up for their primary HNC. Therefore, we believe that the benefit of PET-CT in identifying synchronous primaries currently justifies the prudent work up of incidental avid lesions on PET-CT for HNC.

Nevertheless, the variable, yet high false-positive rates and subsequent implications highlight a need for a standardised pathway for investigating incidental avid lesions in HNC services. Currently, there is scattered (albeit growing) evidence surrounding the outcome of incidental avid lesions in other locations specifically from HNC, leading to variability in practice, both between centres and between staging and response PET-CT scans. A pertinent example from our data is large bowel lesions – 43.6 per cent (17 out of 39) were investigated following response PET compared to just 25 per cent (3 out of 12) in staging PET. This may indicate shifting clinical priority, with more resource focused on the primary HNC at staging PET versus other incidental lesions prioritised once treatment response is complete.

The emphasis that the radiologist places on incidental findings will also influence a clinician’s decision whether to investigate, introducing a potential reporting bias.^10^ Clustering of cancers is often seen due to shared risk factors, such as smoking in both HNC and lung cancer, which should be considered, along with staging and co-morbidities, in the clinical decision of whether to investigate incidental lesions.^9^^,^^11^

Above all, this calls for multi-centre assessment of the prevalence and outcomes of incidental lesions in HNC patients undergoing PET-CT, to first produce a more comprehensive database to reveal varying approaches on a national scale, with the aim of generating of a centralised, data-backed protocol to facilitate the use of a structured, evidence-based approach when counselling HNC patients on incidental lesions in future.

A robust, large-scale database of incidental lesions and their outcomes also represents an investment in future advancements to this research, opening the door to the possibility of using artificial intelligence (AI) to add confidence to the likely outcome of lesions on PET-CT scans, assisting clinicians in decisions about further investigation.

When trying to ascertain whether a lesion is malignant or benign on 18-FDG PET-CT, it is generally accepted that looking at the pattern of the metabolic abnormality is more important than the intensity (SUV, standardised uptake value).^12^ However, the very basis of this study highlights that whilst many lesions have characteristic appearances or ‘metabolic signatures’ that enable confidence in determining their nature from PET-CT alone, many still require further investigation.

In recent years, machine learning, in particular deep learning, has arisen as a potential tool to improve confidence in detection of abnormalities.^13^ Studies investigating the application of radiomics (the conversion of medical images into quantitative high-dimensional data) and deep-learning-based pattern-recognition to HNC have shown modest yet promising results in detecting abnormalities from scans and differentiating malignant and benign outcomes.^14^^,^^15^ These techniques are data-driven, so for this to be a possibility, a large, continuously evolving data set is required to train and continuously ‘feed’ AI models, allowing the program to learn from previous incidental lesions and outcomes, detect patterns and make high-level conclusions about new lesions it is presented with. Currently, the lack of centralised, rigorous data collection in this subject area limits the potential for this technology to be harnessed clinically, but the findings from this research show that with the right expertise, funding and commitment to data collection, this could be an opportunity within our grasp in years to come. As PET-CT becomes more commonplace, both in standard HNC care and large-scale research, such as the ongoing PET-NECK 2 randomised controlled trial (where all patients in the patient-led group receive a PET-CT scan one year post-treatment), the pool from which we can harvest data on incidental lesions only grows, increasing the feasibility of artificial intelligence as an adjunct to decision-making.^16^

### Limitations

The retrospective nature of the study meant that the independent roles of PET and CT components for identifying incidental lesions could not be determined. Often, CT and MRI scans are performed before the PET-CT which could introduce a reporting bias, as radiologists may not state repeated findings in their PET-CT report. In addition, it is difficult to determine the clinical reasoning behind whether a lesion was investigated or not, as digital case notes, multi-disciplinary team (MDT) discussions and test results often lack a detailed explanation of thought process. Finally, finite healthcare resource means that our patients had their PET-CT scans at a single tertiary centre, which is often out of area and combined with barriers to easily accessible health informatics, meant that many patients were excluded as PET-CT scans were either not done or were missing from the case notes or imaging reporting system.

### Comparison with other studies

We were able to find two other studies that focus specifically on incidental findings, including incidental synchronous primaries, in HNC patients undergoing PET-CT. One study by Casselden *et al*. was also conducted in the UK; the other study by Britt *et al*. was from the USA.^8^^,^^17^ Our study covers a larger period than Casselden *et al*. (8 years versus 15 months), with more patients and incidental findings considered. A further study by Beatty *et al*. featured a significant proportion of HNC patients, and their published results allowed calculation of a rate of incidental synchronous primary specific to PET-CT done for primary HNC.^7^ Further details of the characteristics and findings of these studies can be found in Table 5.

**Table 5.** Characteristics and findings of similar studies

First author, year	Location	Study type, level of evidence	Cohort (primary tumour site)	Rate of incidental synchronous primary* (n)	Locations of synchronous primary (n)
Britt, 2017^17^	Wisconsin, USA	Retrospective, level III	Head and neck (100%)	4.1% (12 out of 293)	Thyroid (5), lung (2), gastrointestinal (2), head and neck (1), lymphoma (1), genitourinary (1)
Casselden, 2019^8^	Oxford, UK	Retrospective, level III	Head and neck (100%)	3.2% (3 out of 93)	Oesophagus (1), thyroid (1), lung (1)
Beatty, 2009^7^	Georgia, USA	Retrospective, level III	Head and neck (20%), lung (19%),colorectal (11%), lymphoma (10%), breast (9%), genitourinary (8%), CUP (1%)	11.5% (3 out of 26)	Thyroid (1), genitourinary (1), sarcoma (1)

* Amongst head and neck cancer patients only.

Our overall rate of incidental synchronous primary malignancies was 3.6 per cent; Casselden *et al*. and Britt *et al*. report similar rates of 3.2 per cent and 4.1 per cent, respectively.^8^^,^^17^ The locations of incidental synchronous primaries found in these studies also somewhat reflect those found in our cohort, despite a relatively small number of synchronous primaries across the three studies. Beatty *et al*. reports a considerably higher rate of synchronous malignancy of 11.5 per cent; however, this was amongst 26 HNC patients in a larger cohort of mixed primaries, which is a smaller sample size than our study. The overall rate of incidental synchronous primary on PET-CT for all primary cancers in Beatty *et al*. was 15 per cent.^7^ All of the studies which allow us to calculate a rate of incidental synchronous primary, including our own, are retrospective and represent level 3 evidence, limiting the confidence that clinicians can take from their findings. The findings of this study, along with the other studies above, need to be corroborated by larger-scale studies of a similar nature, to allow conduction of a systematic review of all empirical evidence. This would then permit more robust, evidence-backed protocols to be generated and validated for future use in the management of incidental avid lesions on PET-CT for HNC.

### Clinical applicability and further research

HNC incidence is increasing and with this, more PET-CT scans will be performed, so the issue of incidental lesions will only become more prevalent. This demands research into new technologies to assist radiologists and clinicians in increasing confidence in determining the likely outcome of an incidental avid lesion before investigations are commenced. As alluded to above, AI – machine and deep learning - could be harnessed by training a model in the outcomes of previous incidental lesions, detecting characteristic appearances or patterns on PET-CT and making decisions on the likely outcome of new incidentals it is presented with. In addition, risk calculators could help to reduce false positives by attempting to stratify the likelihood of outcomes based off clinicopathological data, allowing a more informed decision before further investigations. An example for HNC is the HaNC-RC, which predicts the estimated percentage probability of HNC based off demographic and symptom inputs.^18^

**Summary**

- Positron emission tomography-computed tomography (PET-CT) scanning for head and neck cancer (HNC) often reveals incidental avid lesions which may be benign, malignant synchronous primaries or metastatic foci of the known primary
- A lack of standardised pathways for stratifying incidental avid lesions in HNC services leads to variability in whether they are investigated further
- We found that 72.4 per cent of our patients undergoing PET-CT for staging or treatment response had at least one incidental avid lesion on their scan(s), and 33.1 per cent of these were investigated further
- The rate of incidental synchronous primary was 3.6 per cent in our cohort of HNC patients who underwent PET-CT scanning for staging and/or treatment response, which can alter their ongoing management
- The high false-positive rate (incidental avid lesions investigated that turned out to be benign) must be considered when deciding whether to carry out further investigations for incidental lesions.

## Conclusion

PET-CT scanning for HNC can reveal numerous incidental avid lesions which may turn out to be benign, malignant synchronous primaries or metastatic foci of the known primary. Our study demonstrates a rate of incidental synchronous primary of 3.6 per cent in HNC patients who undergo PET-CT scanning for staging and/or treatment response, which is similar to other authors. Despite high false-positive rates, we believe that the benefit of investigating selected incidental PET-CT findings alongside prudent clinical judgement currently outweighs the burden of investigation, as additional diagnoses may impact overall patient management. Above all, these data demonstrate a need for a standardised pathway for stratifying incidental avid lesions in HNC services to ensure consistency of high-quality, evidence-based care for our patients.
